# Abdominal Aortic Intimal Flap Motion Characterization in Acute Aortic Dissection: Assessed with Retrospective ECG-Gated Thoracoabdominal Aorta Dual-Source CT Angiography

**DOI:** 10.1371/journal.pone.0087664

**Published:** 2014-02-04

**Authors:** Shifeng Yang, Xia Li, Baoting Chao, Lebin Wu, Zhaoping Cheng, Yanhua Duan, Dawei Wu, Yiqiang Zhan, Jiuhong Chen, Bo Liu, Xiaopeng Ji, Pei Nie, Ximing Wang

**Affiliations:** 1 Shandong Medical Imaging Research Institute, Shandong University, Jinan, Shandong, P.R. China; 2 Departments of Ultrasound, Shandong Provincial Hospital, Jinan, Shandong, P. R. China; 3 Unit of Periodontology, University of Greifswald, Greifswald, Germany; 4 CT Research Collaboration, Siemens Ltd., China, Beijing, P.R. China; 5 Healthcare Sector, Siemens Ltd., China, Shanghai, P.R. China; Medical University Innsbruck, Austria

## Abstract

**Objectives:**

To evaluate the feasibility of dose-modulated retrospective ECG-gated thoracoabdominal aorta CT angiography (CTA) assessing abdominal aortic intimal flap motion and investigate the motion characteristics of intimal flap in acute aortic dissection (AAD).

**Materials and Methods:**

49 patients who had thoracoabdominal aorta retrospective ECG-gated CTA scan were enrolled. 20 datasets were reconstructed in 5% steps between 0 and 95% of the R-R interval in each case. The aortic intimal flap motion was assessed by measuring the short axis diameters of the true lumen and false lumen 2 cm above of celiac trunk ostium in different R-R intervals. Intimal flap motion and configuration was assessed by two independent observers.

**Results:**

In these 49 patients, 37 had AAD, 7 had intramural hematoma, and 5 had negative result for acute aortic disorder. 620 datasets of 31 patients who showed double lumens in abdominal aorta were enrolled in evaluating intimal flap motion. The maximum and minimum true lumen diameter were 12.2±4.1 mm (range 2.6∼17.4) and 6.7±4.1 mm (range 0∼15.3) respectively. The range of intimal flap motion in all patients was 5.5±2.6 mm (range 1.8∼10.2). The extent of maximum true lumen diameter decreased during a cardiac cycle was 49.5%±23.5% (range 12%∼100%). The maximum motion phase of true lumen diameter was in systolic phase (5%∼40% of R-R interval). Maximum and minimum intimal flap motion was at 15% and 75% of the R-R interval respectively. Intimal flap configuration had correlation with the phase of cardiac cycle.

**Conclusions:**

Abdominal intimal flap position and configuration varied greatly during a cardiac cycle. Retrospective ECG-gated thoracoabdominal aorta CTA can reflect the actual status of the true lumen and provide more information about true lumen collapse. This information may be helpful to diagnosis and differential diagnosis of dynamic abstraction.

## Introduction

Acute aortic dissection (AAD) is a catastrophic disease with fatal outcomes. The overall in-hospital mortality of AAD was 27.4% [1]. The abdominal aortic branch obstruction is the key risk factor for AAD of both type A and type B [2], [3], [4]. Branch obstruction is classified as either “static” or “dynamic” with anatomical features [5]. Treatment decision of AAD has to be made depending on the branch obstruction mechanisms [6]. In the type of dynamic obstruction, the intimal flap spared the origin of the vessel, whereas compressed the true lumen at or above the origin of the aortic branch like a curtain [7]. Previous studies have demonstrated that the position and configuration of intimal flap were correlated with dynamic obstruction [5], [8]. Furthermore, the aorta is a dynamic structure and the intimal flap often oscillates as a result of changing flow conditions [9]. Therefore the diagnostic information of intimal flap dynamics is important.

Conventional CT angiography (CTA) without ECG-gated only can capture the static images of intimal flap and reflect the conformation of the intimal flap at an arbitrary time point. Retrospective ECG-gated CTA allows the phase-resolved cine imaging and visualization. The “four dimension” images can assess the dynamics of the intimal flap movement within a cardiac cycle [9], [10], [11]. In addition, the artifacts of the ascending aorta caused by the cardiac pulsation can be eliminated by this technique. And the accurate anatomical information on entire aorta can lead to a higher diagnostic confidence [12].

However, comparing to the conventional CTA, the retrospective ECG-gated CTA is accompanied by higher radiation dose and longer acquisition time [13]. These disadvantages limit the application of the thoracoabdominal retrospective ECG-gated aorta CTA [14]. As a result, the tailored protocols of the combination of retrospective ECG-gated acquisition of the thoracic aorta with standard protocol for the abdominal aorta were used in the clinical practice [9], [15], [16]. Whereas data acquired from the modified protocols cannot provide the dynamic information on the intimal flap of the abdominal aorta.

With the increased spatial and temporal resolution and tube power of latest CT scanners, the data acquisition of retrospective ECG-gated thoracoabdominal aorta CTA within a single breath hold could be feasible [12], [17]. Furthermore, new technologies on radiation dose reduction have been applied, especially in cardiovascular CTA. These strategies include tube voltage optimization, automatic tube current modulation, ECG-controlled tube current modulation and iterative reconstruction [18], [19], [20], [21], [22]. These integrated technologies have improved the efficiency for radiation dose reduction, and there are some studies on retrospective ECG-gated thoracoabdominal aortic CTA recently [12], [23]. However, to our knowledge, how to evaluate abdominal aortic intimal flap motion with ECG-gated CTA in AAD is still unclear.

The purpose of this study is to evaluate the feasibility of dose-modulated retrospective ECG-gated thoracoabdominal aorta CT angiography (CTA) assessing abdominal aortic intimal flap motion and investigate the motion characteristics of intimal flap in acute aortic dissection (AAD).

## Materials and Methods

### Study Population

This retrospective study was approved by local institutional review board of Shandong Medical Imaging Research Institute, Shandong University, and written informed consent was obtained from each patient before examinations. Patients with suspected or known AAD were referred for retrospective ECG-gated thoracoabdominal aortic CTA. From January 2012 to November 2012, a total of 49 consecutive patients(33 men, 16 women; age range, 30–73 years, mean age ± standard deviation, 49.25±10.4 years)were enrolled in the study. No *β*-blockers or heart-rate-lowering agents were administered before CT data acquisition.

### Retrospective ECG-gated CTA Data Acquisition and Dose Estimation

Retrospective ECG-gated contrast enhanced CTA was performed by dual-source CT (Definition Flash; Siemens Healthcare, Forchheim, Germany) with 2×64×0.6 mm collimation, section acquisition of 2×64×0.6 mm, and a z-flying focal spot technique. The rotation time was 280 milliseconds. The data was acquired in a craniocaudal direction, with the full scanning range extending from the thoracic inlet to the femoral head. All patients were asked to elevate and place arms on the table and hold their breath after deep inspiration during the examination. Tube voltage was adjusted to body mass index (≦25.0 kg/m^2^, 100 kv, n = 26; >25.0 kg/m^2^, 120 kV, n = 23) as previous study [24]. Automatic tube current modulation (Caredose 4D: Siemens Healthcare) [19] was applied using reference mAs setting of 250. ECG-based tube current modulation technique (Adaptive ECG-Pulsing: Siemens Healthcare) [20] was applied using the following parameters. The “phase of interest” was centered at 70% of the R-R cycle. Outside the ECG-pulsing window, tube current was reduced to 20% of the peak tube current. Pitch values were adapted automatically by the lowest expected heart rate on the basis of the longest R-R interval of the last 10 heart beats before the start of data acquisition [25]. The minimum and maximum of pitch values were 0.19 and 0.45, respectively.

Iodinated contrast material (Omnipaque350, GE Healthcare, USA) was injected intravenously through an 18-gauge needle into the upper extremity peripheral vein at a flow rate of 4–4.5 ml/sec (total injected, 100–120 ml), followed by a saline chaser of 30 ml at the same flow rate. This was accomplished by using an electronic power dual injector (Stellant; Medrad, Indianola, PA, USA). Data acquisition was started 5 seconds after the contrast media reached a threshold of 100 Hounsfied unit with a region of interest (ROI) placed in the ascending aorta.

The age, height, and weight of the patients were recorded before the examination,and the average heart rate,scan range, scanning duration, volume CT dose index (CTDIvol), dose length product (DLP) were provided by the scanner. The effective dose estimate was determined by using DLP and appropriate normalized coefficients found in previous report (k = 0.0145 mSv·mGy^−1^.cm^−1^ for chest CT, and k = 0.0153 mSv.mGy^−1^.cm^−1^ for abdominal CT, k = 0.0129 mSv.mGy^−1^.cm^−1^ for pelvic CT) [26]. Because a combination of chest, abdominal, and pelvic acquisitions was performed for thoracoabdominal CTA, the mean conversion coefficients (k = 0.0142 mSv.mGy^−1^.cm^−1^) was used, as previously described [23].

### CT Data Reconstruction

From the ECG-synchronized CT raw datasets, morphological images were generally reconstructed through the entire chest and abdomen at 70% of the R-R interval with 1-mm slice thickness, 0.7-mm increments, I26f reconstruction kernel and 32 cm FOV. Functional imaging was then reconstructed in 5% steps between 0 and 95% of the R-R interval from the ECG with 1-mm slice thickness, 0.7-mm increments, I26f reconstruction kernel and 24 cm FOV. All datasets were reconstructed with sinogram affirmed iterative reconstruction (SAFIRE, Siemens Healthcare, Forchheim, Germany) and a medium strength level of 3 was used in all patients. All datasets were transferred to a Siemens Workstation (Syngo Workplace; Siemens Medical Solutions) for image analysis.

### Assessment of Image Quality and Intimal Flap Movement

Aortic intraluminal attenuation and image noise were assessed as objective image quality parameters. Datasets acquired with full tube current and 20% of the peak tube current was analyzed. Images acquired with 20% of the peak tube current had the similar image quality, so datasets acquired at 25% of R-R interval were chosen randomly for objective image quality analyzed. Then datasets acquired at 25% (acquired with 20% of the peak tube current) and 70% (acquired with full tube current) of R-R interval were assessed. The ROIs were placed in the ascending aorta, descending aorta at the level of the right pulmonary trunk plane and at the level of 2 cm above the celiac trunk ostium. In case of aortic dissection, the ROIs were placed in the true lumen. ROIs used for measure were set as large as possible due to the high variation of the true lumen area during a cardiac cycle. In case of true lumen collapsed completely, the ROIs were placed in the false lumen at the same level. Image noise was assessed as the standard deviation of the mean attenuation in all respective ROIs.

Motion artifact of intimal flap of each phase was assessed as subjective image quality parameters. The criteria for the present of motion artifacts is intimal flap blurred outlines or double range. It was assessed by a consensus reading of two radiologists.

**Figure 1:**
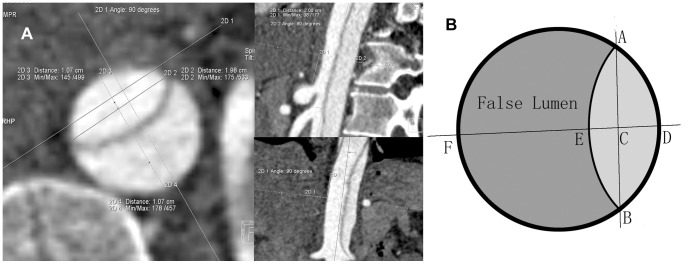
Measurement of lumen short axis diameters A. Double-oblique multiplanar reformations were adjusted perpendicularly to the longitudinal axis of aortic vessel course, at the level of 2 cm distal to the celiac trunk ostium. Outline the true and false lumen short axis diameter manually with the tool of distance measurement. **B.** Schematic illustration of short axis diameter measurement. Connecting the end points A and B of the dissected flap can get the segment AB. The perpendicular line through the mid point C intersects with true lumen wall, dissected flap and false lumen wall at point D, E and F respectively. Segment DE and EF are true lumen and false lumen short axis diameters respectively.

To assess the dynamic changes of intimal flap related to the phase of cardiac cycle, the true lumen short axis diameter (TLD) and the false lumen short axis diameter (FLD) were measured at different phases of a cardiac cycle. TLD and FLD were measured at the level of 2 cm above the celiac trunk ostium by two independent observers with 4 years and 7 years of experience in CTA. To assess the aortic diameter, multiplanar reformation images were taken in a strictly transverse orientation through the abdominal aorta. How TLD was measured was represented in **Fig. 1**. The configuration of the intimal flap was classified as curved toward the false lumen, curved toward the true lumen and flat, as previously described [27]. It was assessed independently by the two observers. In case of disagreement in classifying between the observers, a final decision was obtained by consensus.

### Data Analysis

The method developed by Husmann L et al. [28] was applied in our study, in which all time instances were expressed as percentages of the R-R interval to enable comparisons of intimal flap motion amplitude for all patients. The motion amplitude of intimal flap was quantified by the change of the TLD at every 5% R-R interval. It was expressed as the absolute value of relative change between the TLD (RCTLD) of adjacent phases of R-R interval. With this approach, for example, intimal flap motion amplitude of the time interval 20% refers to the absolute difference of TLD between 15% and 20% of the R-R interval. On the basis of measurements, the following variables were obtained:

(1) The maximum change of TLD (MCTLD) and relative decrease of TLD (RDTLD) during a cardiac cycle: MCTLD was defined as the difference between individual maximum TLD (TLDmax) and minimum TLD (TLDmin) over a cardiac cycle; RDTLD was defined as the ratios of MCTLD to TLDmax. MCTLD reflect the range of intimal flap motion and RDTLD reflect the extent of TLDmax decreased during a cardiac cycle. (2) RCTLD and maximum motion phase (MMP) of TLD over a cardiac cycle: RCTLD defined as the absolute difference between the TLD of adjacent time intervals, which reflects the change of TLD at a given percentage of time intervals. MMP was defined as the phase of R-R interval where the maximum RCTLD was at. The design formulas were as follow:
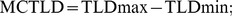


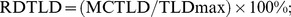


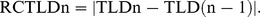



Where “n” represents a given phase of the R-R interval and “(n−1)” represents the previous phase.

### Statistical Analysis

Quantitative variables were presented as the mean ± standard deviation (ranges). Objective image quality of datasets acquired at 25% and 70% of R-R interval display normal distribution. They were compared with the paired two-tailed Student’s t-test. A value of *p*<0.05 was considered statistically significant. Interobserver variation of diameter measurement was evaluated by using the Bland-Altman analysis [29]. Interobserver agreement on classifying the intimal flap configuration was calculated before consensus reading by using Kappa statistics. A kappa value of more than 0.81 corresponded to excellent interobserver agreement, with values of 0.61–0.80 corresponding to good agreement [24]. TLD, FLD measurements were averaged for both observers. TLD, FLD displayed normal distribution, but the RCTLD did not. Therefore, RCTLD was log transformed before analysis to meet assumptions of normality. Bonferroni post-hoc tests were used for multiple pair wise comparisons of FLD, TLD and RCTLD within time intervals. A value of *p*<0.05 was considered statistically significant. All statistical analyses were performed with statistical software SPSS 17.0 (SPSS, Chicago, IL, USA).

## Results

All retrospective ECG-gated thoracoabdominal aortic CTA examinations were successful. In these 49 patients, 37 had AAD, 7 had intramural hematoma, and 5 had negative result for acute aortic disorder. Onset time of AAD was from 2 hours to 12 days. According to DeBakey classification [30], 20 of the 37 (54.1%) patients were type I, 3 of the 37 (8.1%) patients were type II, and 14 of the 37 (37.8%) were type III. 620 datasets of 31 patients (both type I and type III) who showed double lumens in abdominal aorta were enrolled in evaluating intimal flap motion. The data acquisition characteristics and radiation doses of 49 patients were listed in **Table 1**. The CTDIvol, DLP, and effective dose were 16.1±6.8 (7.6∼33.6) mGy, 896.4±373.4 (450.1∼1850.1) mGy.cm, and 12.7±5.3 (6.4∼26.3) mSv respectively.

**Table 1:** Data acquisition characteristics and radiation exposure estimates.

Characteristic	Result
BMI (kg/m^2^ )	24.64±3.36(18.20∼35.33)
Heart rate (beats/min)	75.5±13.3(50∼105)
Pitch	0.30±0.05(0.19∼0.45)
Kv* 120	23
100	26
Scanning duration (sec)	15.8±2.8 (9.3∼22.8)
Scanning range (mm)	578.8±36.2 (499∼706)
CTDI_vol_ (mGy)	16.1±6.8(7.6∼33.6)
Dose-lengthproduct(mGy · cm)	896.4±373.4(450.1∼1850.1)
Estimated effective radiation(mSv)	12.73±5.3(6.4∼26.3)

Note.–Data are means ± standard deviations. Ranges are in parentheses.

* The tube voltage was 100 kV for patients with a BMI of less than 25 kg/m^ 2^ and 120 kV for those with a BMI of at least 25 kg/m^ 2^. ***BMI*** = Body Mass Index, ***CTDI_vol_*** = volume CT Dose Index.

### Objective and Subjective Image Quality

Datasets acquired at 70% R-R interval had preferable image quality due to its lower image noise and less intimal flap motion artifacts. Image noise was significantly lower in datasets of 70% R-R interval compared with datasets of 25% R-R interval in all measurements (ascending aorta, 26.8±6.4 vs 48.9±5.2, p<0.01; descending aorta, 23.2±5.5 vs 46.2±5.8, *p*<0.01; abdomen aorta, 24.4±5.1 vs 47.3±5.4, *p*<0.01). There were no statistically significant differences in intraluminal contrast attenuation between both datasets in all measurements (ascending aorta, 386.9±63.6 Hu vs 382.4±65.1 Hu, *p*>0.05; descending aorta, 381.4±55.3 Hu vs 371.9±58.8 Hu, *p*>0.05; abdomen aorta, 377.6±60.0 Hu vs 376.0±62.4 Hu, *p*>0.05).

**Figure 2:**
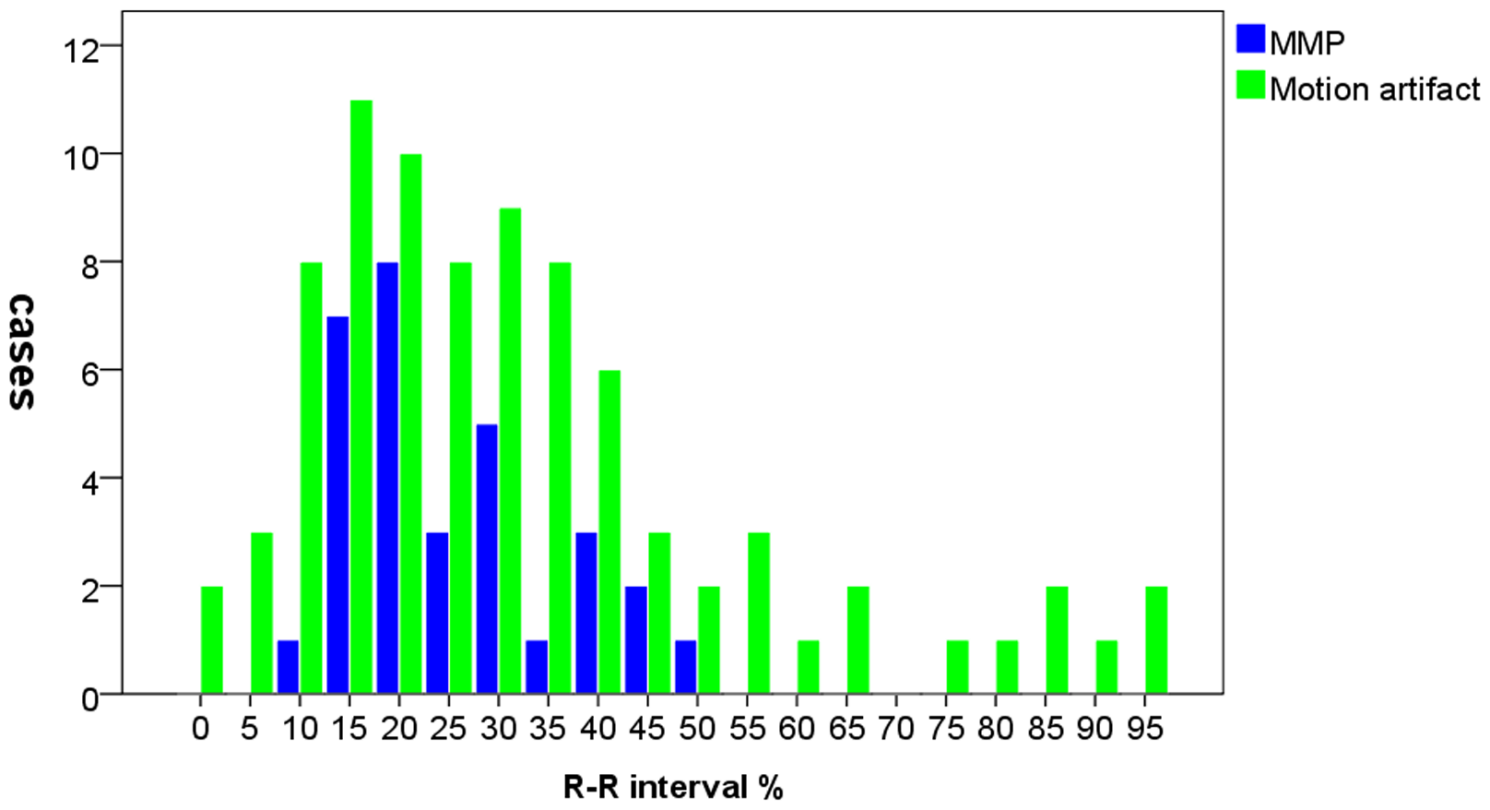
The frequency distributions of MMP and intimal flap motion artifacts during a cardiac cycle. The MMP of all cases was at systolic phase (5%∼40% of R-R interval) and the peak was found at 15% of the R-R interval. Most intimal flap motion artifacts were founded at systolic phase. Datasets acquired at 70% R-R interval had no intimal flap motion artifacts.

The frequency distributions of intimal flap motion artifacts during a cardiac cycle were represented in **Fig. 2**. 83 of 620 (13.4%) intimal flaps had motion artifacts. 65 of 83(78.3%) intimal flaps motion artifacts were at systolic phase (0%∼40% of R-R interval) and there was a peak at 15% of the R-R interval. Datasets acquired at 70% R-R interval had no intimal flap motion artifacts.

### Interobserver Variation Assessment

The Bland-Altman analysis of interobserver variation of diameter measurements demonstrated good results. The measurement error of TLD and FLD were 0.085 and 0.078 respectively, whereas the 95% limits of agreement were1.630 to −1.460 and 1.410 to −1.254 mm respectively. Overall interobserver agreement on intimal flap configuration classifying was excellent (*k* = 0.93).

### Motion Characterization of Intimal Flap

**Figure 3:**
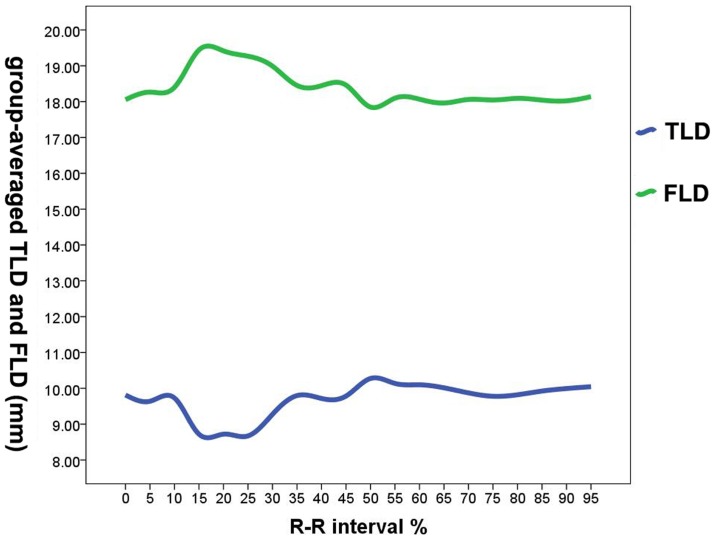
Time courses of TLD and FLD during the R-R interval. Group-averaged TLD, FLD of each phase were plotted against time in percentages of R-R interval. Group-averaged FLD was larger than group-averaged TLD in every phase. Although there was no statistically significant difference in R-R intervals, a peak for group-averaged FLD and a trough for group-averaged TLD were found in 15% of R-R interval.

Time courses of group-averaged TLD and FLD were represented in **Fig. 3**. Both TLD and FLD changed during the cardiac cycle and their dynamic behaviors were highly variable for each patient. Peak of group-averaged FLD (19.44±4.07 mm) and trough of group-averaged TLD (8.67±5.16 mm) were found at 15% of the R-R interval. However, there were no significant statistical differences comparing to other R-R intervals (p>0.05).

The range and means ± standard deviations of the TLDmax, TLDmin, MCTLD and RDTLD in all patients were listed in **Table 2** **.** The range of intimal flap motion in all patients was 1.8∼10.2 mm, means ± standard deviations was 5.5±2.6 mm. TLDmax can decrease up to 100% (mean: 49.5%) during a cardiac cycle. The frequency distributions of MMP during a cardiac cycle were represented in **Fig. 2**. All of the MMP were at systolic phase (5%∼40% of R-R interval) and there was a peak at 15% of the R-R interval.

**Table 2:** True lumen diameter characteristics.

characteristic	median	range
TLDmax(mm)	12.2±4.1	2.6∼17.4
TLDmin(mm)	6.7±4.1	0∼15.3
MCTLD(mm)	5.5±2.6	1.8∼10.2
RDTLD	49.5%±23.5%	12%∼100%

Note.–***TLDmax*** = maximum True Lumen Diameter, ***TLDmin*** = minimum True Lumen Diameter, ***MCTLD*** = Maximum Change of True Lumen Diameter, ***RDTLD*** = Relative Decrease of maximum True Lumen Diameter.

**Figure 4:**
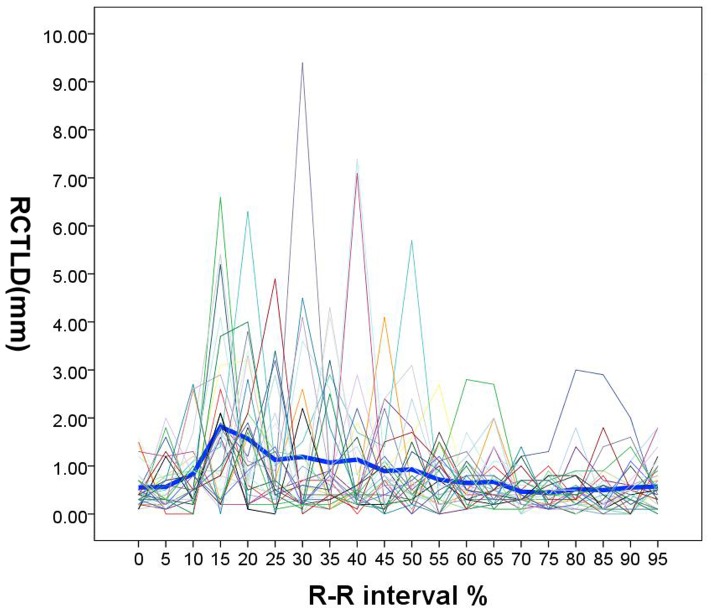
Time courses of RCTLD during the R-R interval. Individual (light line) and group-averaged (dark line) RCTLD were plotted against time in percentages of R-R interval. Despite high inter-individual variation, group-averaged RCTLD showed clear biphasic pulsatility in 10%–25% of R-R intervals.

Time courses for individual and group-averaged RCTLD were represented in **Fig. 4**. Although the dynamic behaviors of intimal flap varied greatly in each patient, the group-averaged intimal flap motion was synchronized to the heart pulsation. Peak intimal flap motion amplitude (1.82±1.69 mm) was found at 15% of the R-R interval; group-averaged intimal flap motion amplitude in this interval was higher than those at any other time intervals (0%–10% and 55%–95%: *p*<0.05; 20%–50%: *p*>0.05). The minimum intimal flap motion amplitude was found at 75% (0.440±0.31 mm) of the R-R interval, and second minimum (0.464±0.31 mm) was found at 70% of the R-R interval. Intimal flap motion amplitude in these two R-R intervals was less than that in other R-R intervals (15%, 20%: *p*<0.05; 0%–10% and 25%–95%: *p*>0.05).

### Intimal Flap Configuration

3 types of intimal flap configurations were found in every R-R intervals. 312 of 620(50.3%) intimal flaps curved toward the false lumen, 226 of 620(36.5%) intimal flaps curved toward the true lumen, and 82 of 620(13.2%) intimal flaps were flat**.** However their distribution within the time interval was different. Initial flap curved toward the false lumen at 0%∼5% and 35%∼95% of the R-R interval in over half the samples. This tendency was reversed at 10∼30% of R-R interval. Intimal flap curved toward the true lumen (64.5%) or were flat (22.6%) at 15% and 20% of the R-R interval. Only 4 of 31 (12.9%) intimal flap curved toward the false lumen at 15% and 20% of the R-R interval.

## Discussion

By exploiting the currently available options for dose reduction, our retrospective ECG-gated CTA protocol of the whole aorta could maintain radiation doses within acceptable limits, according to the reference values of the International Commission on Radiological Protection [31] (DLP of 650, 780, 570 mGy.cm and CTDIvol of 30, 35, 35 given for routine thorax, abdominal and pelvic scan protocols, respectively). The estimated median effective dose was 12.73±5.30(rang, 6.39∼26.27) mSv, which was similar with normal whole chest retrospective ECG-gated CTA (triple-rule-out CTA) [32] and prospective ECG-gated whole aorta CTA [33]. However, both the latter cannot be used for dynamic abdominal aortic intimal flap assessment and may suffer from motion artifacts in abdominal aorta.

Other ECG-gating techniques were recently introduced in whole aorta CTA with lower radiation dose. Prospective ECG-gating sequential data acquisition was introduced for the thoracic aorta or whole aorta CTA, resulting in further dose reduction [24], [33]. In these studies, data acquisition time during a cardiac phase was set to a narrow window without additional padding time for further reconstructions. Then it could not reconstruct datasets of whole cardiac phase and assess the aortic intimal flap oscillation. If we expand the acquisition time window to the whole cardiac phase to assess intimal flap motion, the radiation dose will increase significantly. In addition, longer acquisition time is another defect of prospective ECG-gating sequential data acquisition. It will increase the contrast load [34], and the patients may be unable to hold their breath due to the large scan range in our study. High-pitch technique combined with prospective ECG triggering can provide volumetric data at a prespecified phase in the cardiac cycle and reduce radiation dose significantly [35]. However, data acquired by ECG-gated high-pitch CTA can not evaluate the motion of intimal flap because no slice overlap is used.

**Figure 5:**
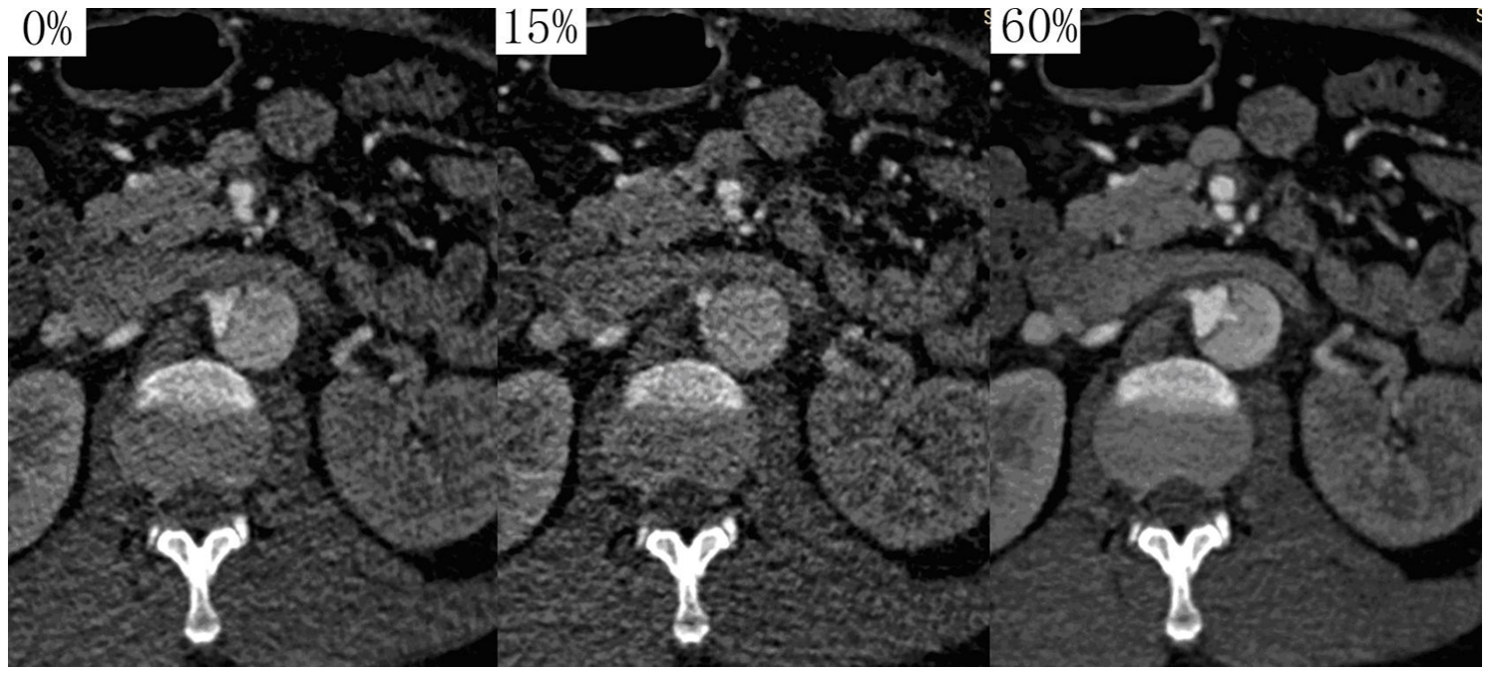
Images in different phases demonstrated different anatomical features. At the right renal artery origin, 0% and 15% datasets could not demonstrate reentry tear, but 60% datasets can demonstrate reentry tear clearly. It was worthy to note that true lumen was completely collapsed and the flap nearly was invisible in 15% R-R interval. However, it was of reasonable caliber in 60% R-R interval.

The ECG-controlled tube current modulation technique plays an important role in the radiation dose reduction while allowing the full phases data acquisition to analyze the aortic flap movement and determine the optimal phase in our study. It has been described in previous studies that the aortic root and ascending thoracic aorta have the greatest motion during systole and have the least motion at mid to late diastole [36]. To acquire motion-free images of ascending aorta, we centered the “phase of interest” at 70% of the R-R cycle. To maintain radiation doses “as low as reasonably achievable”, the pulsing window was strictly chosen to a tailored level with the full tube current exposure whilst the outside pulsing window with 20% of the full tube current which allowed for further image reconstructions in the study. Compared with full tube current datasets, images acquired with 20% of the peak tube current had inferior image quality for the increase image noise. But it could meet the requirements for the measurement of lumen diameter and assessment of configuration. Because in the interobserver analysis, both the Bland-Altman analysis of lumen diameter and *k* test of classifying initial flap configuration demonstrated excellent interobserver agreement. Moreover, these datasets were able to provide additional information. For instance they were helpful for demonstrating entry tears and reentry tears because both tears might not be identified clearly in some phases due to motion artifacts or shrinking of the intimal flap **(** **Fig. 5** **)**.

Our study demonstrated peak intimal flap motion was at 15% of the R-R interval. Centering the “interest phase” at 70% of the R-R cycle not only eliminates the ascending aorta motion artifact,but also avoids the MMP of intimal flap, allowing for motion-free images of the abdominal intimal flap in most cases. Thus, centering the “interest phase” at 70% or 75% of the R-R cycle may be the optimal ECG pulsing window for retrospective thoracoabdominal aorta CTA in AAD.

The intimal flap motion was related to blood pressure change of the two channels. Our study demonstrated the MMP was at systolic phase and maximum intimal flap motion was found at 15% of the R-R interval. This indicated that the transmural pressure gradient on the intimal flap was the highest at systolic phase. The flap would always curve to the lumen with lower pressure. So the direction of flap curvature was pivotal in estimating transmural pressure gradient on the intimal flap. Our study showed that transmural pressure gradient on the intimal flap varied and the variation was related to the phase of cardiac cycle. During middle and later systolic phase, the false lumen pressure was higher than or equivalent to true lumen pressure in most patients. However, during diastolic phase and early systolic phase, the false lumen pressure was lower than true lumen pressure in more than half patients. Only 4 of 31 (12.9%) intimal flap curved toward the false lumen at 15% and 20% of the R-R interval during middle and later systolic phase. This variation may be related to the different anatomic configurations of tear size, number and location [37], [38].

**Figure 6:**
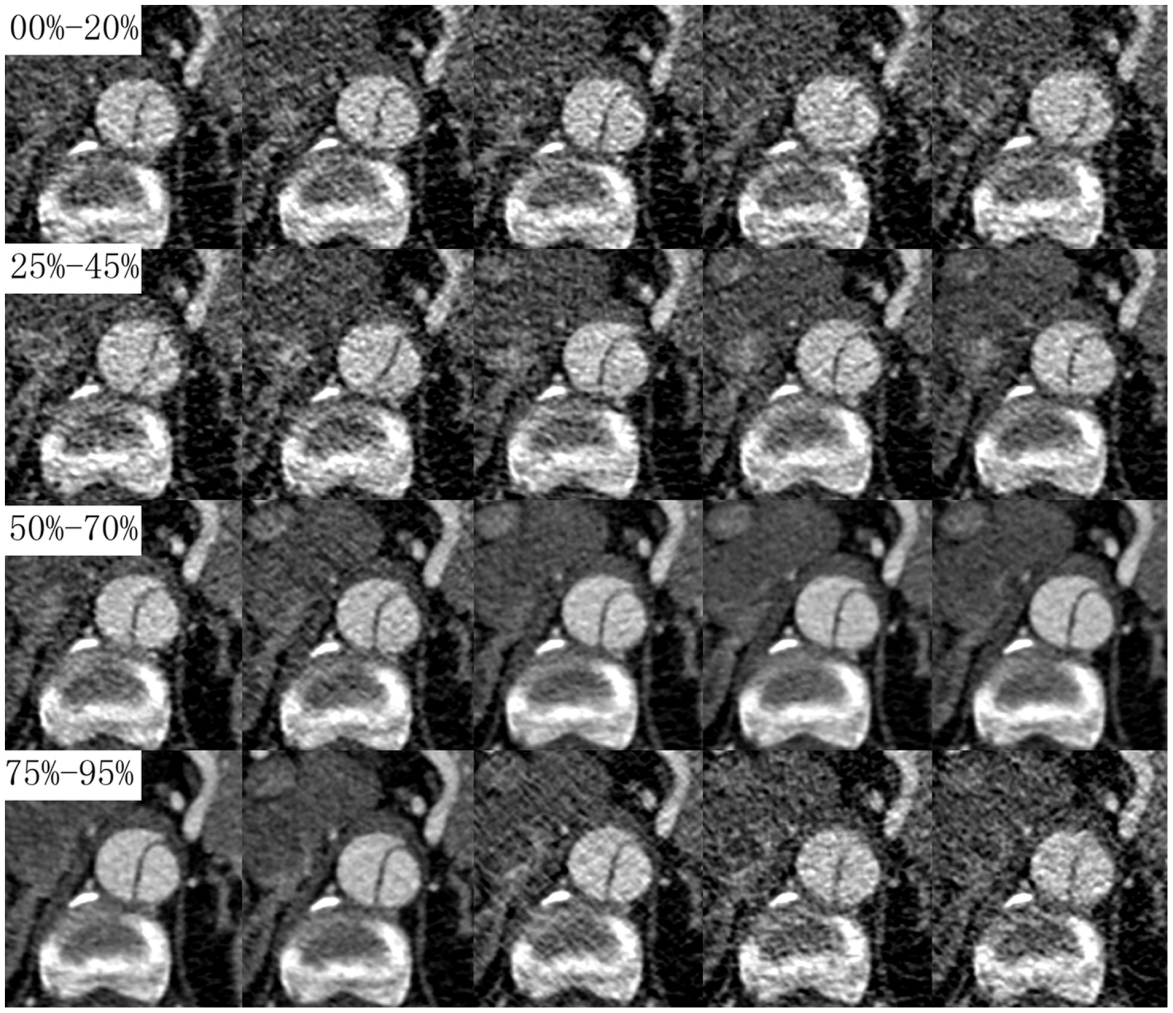
Transverse CT images of different phases at the same level. The intimal flap configuration, position and area of the true and false lumens were extremely variable during a cardiac cycle at the same level.

**Figure 7:**
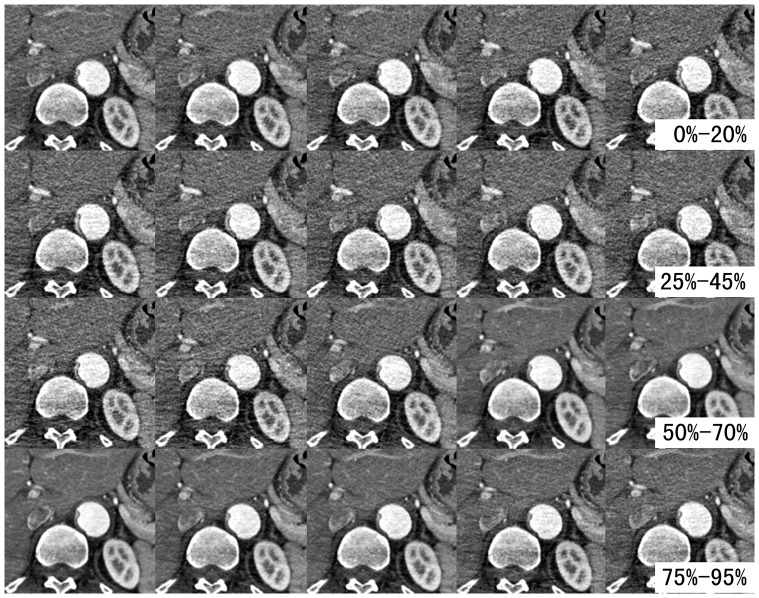
True lumen completely collapses during the cardiac cycle. 0–95% datasets showed that the true lumen was completely collapsed. The position of internal flap was changed mildly, but the configuration of intimal flap was not changed in different R-R intervals.

Previous studies demonstrated that the position and configuration of intimal flap were correlated with dynamic obstruction [5], [8]. Our study demonstrated that the intimal flap oscillated to a large extent during a cardiac cycle. The intimal flap’s configuration, position, and then the area of the true and false lumens varied extremely at the same level **(** **Fig. 6** **and Movie S1)**. In some cases, the true lumen even collapsed partially. A false lumen could completely fill the aorta and obliterate the true lumen in some certain R-R intervals, whereas in other R-R intervals both lumens were of reasonable calibers **(** **Fig. 5** **)**. In such cases, the estimation of the true lumen collapse will conflict with images reconstructed from different R-R intervals. However, in other cases, the true lumen collapsed completely **(** **Fig. 7** **)**. In such cases, the position of internal flap was changed mildly, but the configuration of intimal flap was not changed in different R-R intervals. Conventional static images acquired by non-gated CT often provide anatomical features (such as the position of intimal flap) at an arbitrary location across the cardiac cycle. Then potential errors may occur when we use static images to assess whether the true lumen collapses or not.

Different from static CT images, “four dimension” images acquired with prospective ECG-gated can provide dynamic information of abdominal aortic intimal flap. They can tell whether complete or partial collapse existed in the true lumen. According to the definition of dynamic abstraction, true lumen collapse is the key factor to diagnose dynamic abstraction [5]. Then this information may be helpful to diagnosis and differential diagnosis of dynamic abstraction.

### Limitations

In this feasible study, we only assessed the intimal flap motion at the level of top 2 cm to celiac trunk ostium. Because intraaortic hemodynamic is greatly variable in different regions of the aorta [11], [38], the intimal flap motion in different regions may have different details. In consequence, more levels should be analyzed in future studies, such as at every branch origin plane.

## Conclusions

Dose-modulated retrospective ECG-gated thoracoabdominal aorta CTA can assess the abdominal intimal flap motion in acute AAD in addition to the primary diagnostic question. Abdominal intimal flap position and configuration varied greatly during a cardiac cycle and the variation was related to the phase of cardiac cycle. Retrospective ECG-gated thoracoabdominal aorta CTA can reflect the actual status of the true lumen and provide more information about true lumen collapse. This information may be helpful to diagnosis and differential diagnosis of dynamic abstraction.

## Supporting Information

Movie S1 **Intimal flap configuration, position changed during a cardiac cycle.** The intimal flap configuration, position and area of the true and false lumens were extremely variable during a cardiac cycle at the same level and the motion of intimal flap in different regions had different details.
(MPG)
